# Predicting glucocorticoid effectiveness in thyroid eye disease: combined value from serological lipid metabolism and an orbital MRI parameter

**DOI:** 10.1530/ETJ-23-0109

**Published:** 2024-02-14

**Authors:** Haitao Zhang, Hao Hu, Yueyue Wang, Xinjie Duan, Lu Chen, Jiang Zhou, Wen Chen, Weizhong Zhang, Xiaoquan Xu, Huanhuan Chen

**Affiliations:** 1 Department of Endocrinology, The First Affiliated Hospital of Nanjing Medical University, Nanjing, China; 2 Department of Radiology, The First Affiliated Hospital of Nanjing Medical University, Nanjing, China; 3 Department of Ophthalmology, The Friendship Hospital of Ili Kazakh Autonomous Prefecture Ili & Jiangsu Joint Institute of Health, Ili, China; 4 Department of Ophthalmology, The First Affiliated Hospital of Nanjing Medical University, Nanjing, China

**Keywords:** thyroid eye disease, glucocorticoid therapy, effectiveness, serological lipid metabolism, MRI

## Abstract

**Purpose:**

The aim was to determine the combined value of serological lipid metabolism and an orbital MRI quantitative parameter in predicting the effectiveness of glucocorticoid (GC) therapy in patients with thyroid eye disease (TED).

**Methods:**

This study retrospectively enrolled 46 patients with active and moderate-to-severe TED (GC-effective group, *n* = 29; GC-ineffective group, *n* = 17). Serological lipid metabolism, the orbital MRI-based minimum signal intensity ratio of extraocular muscles (EOM-SIR_min_), as well as other clinical parameters before GC therapy were collected and compared between the two groups. Multivariate logistic regression and receiver operating characteristic curve analysis were adopted to identify independent predictable variables and assess their predictive performances.

**Results:**

Compared to the GC-ineffective group, the GC-effective group showed lower serum total cholesterol levels (*P* = 0.006), lower serum low-density lipoprotein cholesterol levels (*P = *0.019), higher EOM-SIR_min_ values (*P* = 0.005), and shorter disease durations (*P* = 0.017). Serum total cholesterol and EOM-SIR_min_ were found to be independent predictors of GC-effective TED through multivariate analysis (odds ratios = 0.253 and 2.036 per 0.1 units, respectively) (both *P* < 0.05). The integration of serum total cholesterol ≤4.8 mmol/L and EOM-SIR_min_ ≥ 1.12 had a better predictive efficacy (area under the curve, 0.834) than EOM-SIR_min_ alone, with a sensitivity of 75.9% and a specificity of 82.4% (*P* = 0.031).

**Conclusion:**

Serological lipid metabolism, combined with an orbital MRI-derived parameter, was a useful marker for predicting the effectiveness of GCs in patients with active and moderate-to-severe TED.

## Introduction

Thyroid eye disease (TED) is a specific autoimmune condition associated with inflammation of the thyroid, orbit, and periorbital tissues (1, 2) that often endangers the patient’s vision and appearance (3). TED patients commonly progress through two different stages, transitioning from an initial active phase of continuous inflammation to an inactive phase of inflammation extinction, with the disease course generally lasting about 18–24 months (3, 4, 5). Intravenous high-dose glucocorticoids (GCs), the current first-line choice for patients with active and moderate-to-severe TED, have potent immunosuppressive effects and can rapidly control the inflammatory phase to improve the acute symptoms (6, 7). However, in clinical practice, GC therapy is ineffective in >20% of cases (6, 8), and the clinical symptoms of patients with GC-unresponsive TED cannot be significantly improved. In addition, patients may also be affected by the side effects of GCs, such as infection, hyperglycemia, osteoporosis, Cushing’s syndrome, cardiovascular and cerebrovascular complications, among others (9). GC-effective TED patients could be treated with GCs, and those who are unresponsive should choose other therapies as soon as possible to avoid delays in treatment. Therefore, it is crucial to predict the response of TED patients to GC therapy before initiating treatment. Through this study, we aimed to obtain a simple and accurate method to help predict the response to intravenous GC therapy.

The detection of serological lipid metabolism parameters, mainly including serum total cholesterol (TC), low-density lipoprotein cholesterol (LDL-C), high-density lipoprotein cholesterol (HDL-C), and triglyceride (TG), is clinically simple and readily possible (10). Lipid metabolism plays a crucial role in maintaining the normal life activities of the body. Recent studies have suggested that hypercholesterolemia may be a new risk factor for developing TED (6, 11, 12, 13). One study found that high baseline serum LDL-C levels may reduce the efficacy of intravenous GC therapy in patients with active and moderate-to-severe TED (14). Hypercholesterolemia was suggested to potentially increase the immune process of TED and promote orbital fibrosis and remodeling by inducing chronic inflammatory conditions (14). However, the assessment of serum biochemical indicators may be affected by aspects of physiology, metabolism, and the environment to a certain extent. Therefore, other objective, stable, and effective assessment methods are required.

In recent years, orbital MRI has been used increasingly in the clinical diagnosis and evaluation of TED. Studies have suggested that the volume and thickness of extraocular muscles (EOMs) correlate with the GC therapy response (15, 16, 17). Further research, based on the signal intensity (SI) of EOMs, has suggested that the SI ratio (SIR) of EOMs (EOM-SIR) could better predict the effectiveness of GC therapy (16, 17). All these findings indicate an intrinsic link between the response to GC therapy and the degree of orbital inflammation and fibrosis in TED patients (16, 17).

Accordingly, the objectives of this study were (i) to determine whether the combination of serological lipid metabolism and orbital MRI parameters in patients with active and moderate-to-severe TED could help predict the GC therapy response and (ii) to explore whether the integration of both could further improve the predictive power compared to using a single indicator alone.

## Materials and methods

### Patients

**Figure 1:**
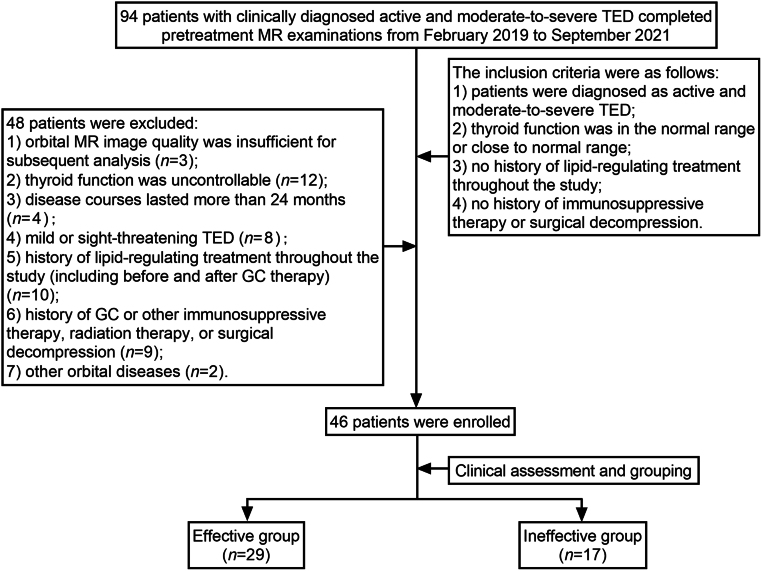
STARD diagram of patient enrollment. TED, thyroid eye disease; MR, magnetic resonance; GC, glucocorticoid.

This study was conducted in accordance with the Declaration of Helsinki and approved by the institutional review board (No. 2019-SR-416). The need to collect informed consent requirement was waived due to the retrospective study design. From February 2019 to September 2021, 94 patients with clinically diagnosed active and moderate-to-severe TED completed pretreatment MRI examinations at our hospital. All TED patients were diagnosed according to Bartley criteria (18). The inclusion criteria of our study were as follows: (i) patients diagnosed with active and moderate-to-severe TED; (ii) thyroid function within the normal range or close to the normal range; (iii) no history of lipid-regulating treatment throughout the study; (iv) no history of immunosuppressive therapy or surgical decompression. And the exclusion criteria were as follows: (i) orbital MRI scan quality was insufficient for subsequent analysis; (ii) thyroid function was uncontrollable; (iii) disease courses lasted >24 months; (iv) mild or sight-threatening TED; (v) history of lipid-regulating treatment throughout the study (including before and after GC therapy); (vi) history of GC or other immunosuppressive therapy, radiation therapy, or surgical decompression; and (vii) other orbital diseases. Forty-eight patients were excluded, and 46 patients (average age: 47.1 ± 10.5 years; male/female: 22/24) were finally enrolled. The STARD diagram of patient enrollment is shown in Fig. 1. According to the consensus of the European Group on Graves’ Orbitopathy (EUGOGO), all patients received recommended intravenous GC therapy with a cumulative dose of 4.5 g methylprednisolone for 12 weeks (0.5 g each time for the first 6 weeks, then 0.25 g each time for the next 6 weeks) (19).

### Clinical evaluation

The period between the onset of TED-related ophthalmic symptoms and the time of the last orbital MRI scan before treatment was designated as the ophthalmopathy duration. The clinical activity of the ophthalmopathy was assessed according to the modified 7-point clinical activity score (CAS), with CAS ≥3 points indicating active disease and CAS <3 points indicating inactive disease (20). The EUGOGO classification was applied to assess the severity of the ophthalmopathy, which was grouped into three levels, as follows: mild, moderate-to-severe, and sight-threatening TED (6). The parameter information of the side with the larger value in the binocular data of each patient was recorded. Patients’ history of antithyroid drugs or thyroid hormone replacement therapy was also collected.

All patients with TED were examined by the same ophthalmologist and endocrinologist both before treatment and 6 months after completing GC therapy. GC therapy was considered effective if (i) CAS decreased by ≥2 points and CAS <3 points and (ii) at least one of the following symptoms had improved without deteriorating others: degree of proptosis decreased by ≥2 mm; eyelid width decreased by ≥2 mm; Gorman score declined by at least one class; and visual acuity improved by ≥1 Snellen line. GC therapy was considered ineffective if CAS decreased by <2 points or the disease remained in the active stage (CAS ≥ 3 points) after GC therapy (8, 21, 22, 23).

### Serological lipid metabolism parameter collection

Fasting serological lipid metabolism parameters (including TC, LDL-C, HDL-C, and TG) of all enrolled patients were collected from the electronic medical records system within 1 week before GC therapy. Normal reference ranges were as follows – TC: 3.00–5.70 mmol/L; LDL-C: 2.60–4.10 mmol/L; HDL-C: 1.03–1.55 mmol/L; and TG: 0.00–2.25 mmol/L.

### Image acquisition and analysis

The enrolled patients were all examined using a 3.0-Tesla MRI system (Magnetom Skyra; Siemens Healthcare, Erlangen, Germany) with a 20-channel head coil. All patients were guided to take a supine position and keep their eyes closed during the scan. Coronal T2-weighted imaging (T2WI) with fat saturation was performed. Specific parameters included the following: acquisition matrix, 224 × 320; field of view, 180 × 180 mm^2^; slices, 18; slice thickness, 3.5 mm; and repetition/echo time, 4000/75 ms.

**Figure 2:**
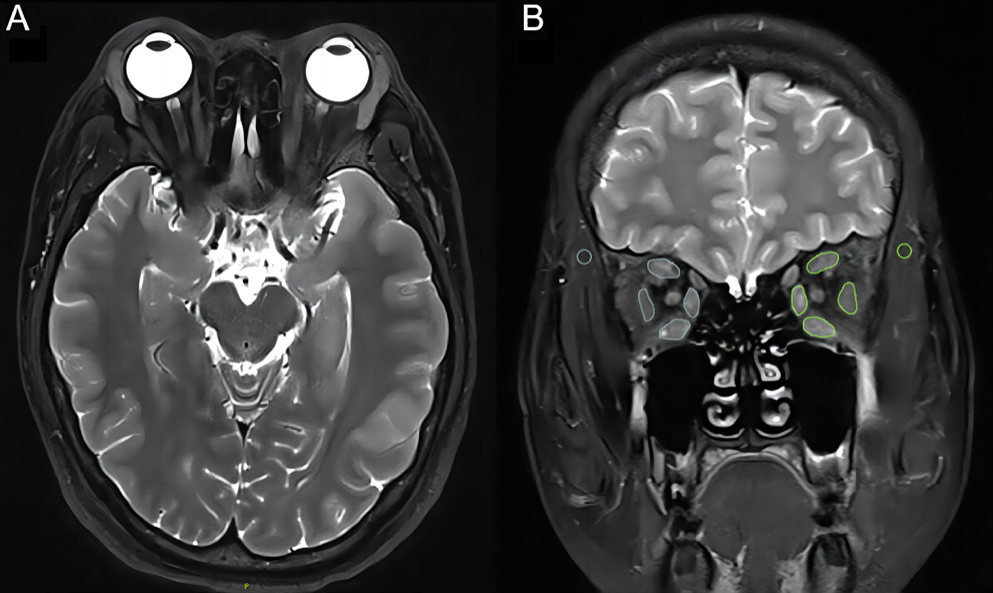
Method of ROI placement for EOMs and temporal muscle. Axial (A) and coronal (B) T2-weighted images with fat saturation of a 27-year-old female patient with TED. The method of drawing ROI is shown in (B). TED, thyroid eye disease; ROI, region of interest.

Based on the results of coronal T2WI with fat saturation, the largest layer of EOMs was selected (usually the third retrobulbar layer). After manually delineating polygon regions of interest (ROIs) on the four EOMs (superior, inferior, medial, and lateral), the minimum SIs of the ROIs of the EOMs in each eye were recorded and a circular ROI (approximately 5–10 mm^2^) was drawn on the ipsilateral temporal muscle (Fig. 2). The absolute SI was then standardized to the SIR using the following formula: SIR = SI_EOM_/SI_ipsilateral temporal muscle_. Finally, the smaller value of the binocular minimum EOM-SIR (EOM-SIR_min_) was recorded (16, 17).

The above imaging parameters were measured independently by two radiologists (reader 1 had 7 years of experience in head and neck radiology; reader 2 had 11 years of experience in head and neck radiology). The intraclass correlation coefficient (ICC) was calculated to evaluate interreader reproducibility.

### Statistical analysis

The normal distribution of continuous variables was analyzed with the Kolmogorov–Smirnov test. Normally distributed data were described using mean ± s.d.values, and an independent samples *t*-test was used for comparison. Nonnormally distributed data were expressed as median (interquartile range) values, and the Mann–Whitney *U* test was used for comparison. Categorical variables were analyzed using the chi-squared test. The differences in serological lipid metabolism and the orbital MRI parameter (EOM-SIR_min_) between the GC-effective and -ineffective groups were evaluated. Other clinical variables – such as age; sex; disease duration; smoking history; body mass index (BMI); thyroid-stimulating hormone receptor antibody (TRAb); the history of thyroid medication; the time since normal-range serum-free triiodothyronine (FT_3_); and thyroid function before, during, and after treatment (including FT_3_, free thyroxine (FT_4_), and thyroid-stimulating hormone) – were also collected and compared. Ophthalmic findings, including visual acuity, intraocular pressure, exophthalmos, eyelid width, the presence of diplopia, and binocular CAS, were also collected for evaluation. Then, those variables with a *P* < 0.10 in the univariate analysis were included in the multivariate logistic regression analysis to identify the independent predictors of GC therapy response. Multicollinearity between variables existed when the variance inflation factor (VIF) was >10. Logistic regression was used to integrate the identified predictors. Receiver operating characteristic (ROC) curve analysis was employed to assess the predictive power of the identified variables and their integration.

Inter-observer reproducibility of orbital MRI quantitative measurement was evaluated using ICC with 95% CIs, and bidirectional methods with random evaluator assumptions were used. ICCs <0.40 indicate poor reproducibility, those 0.40–0.60 suggest moderate reproducibility, those 0.61–0.80 indicate good reproducibility, and those ≥0.81 suggest excellent correlation, respectively. *P* < 0.05 was considered statistically significant. Data management and analysis were performed using SPSS software (v.25.0; IBM Corporation) and MedCalc software (v.20.0; MedCalc Software Ltd, Ostend, Belgium).

## Results

### Comparison of clinical variables, including serological lipid metabolism parameters, between the GC-effective and -ineffective groups

**Figure 3:**
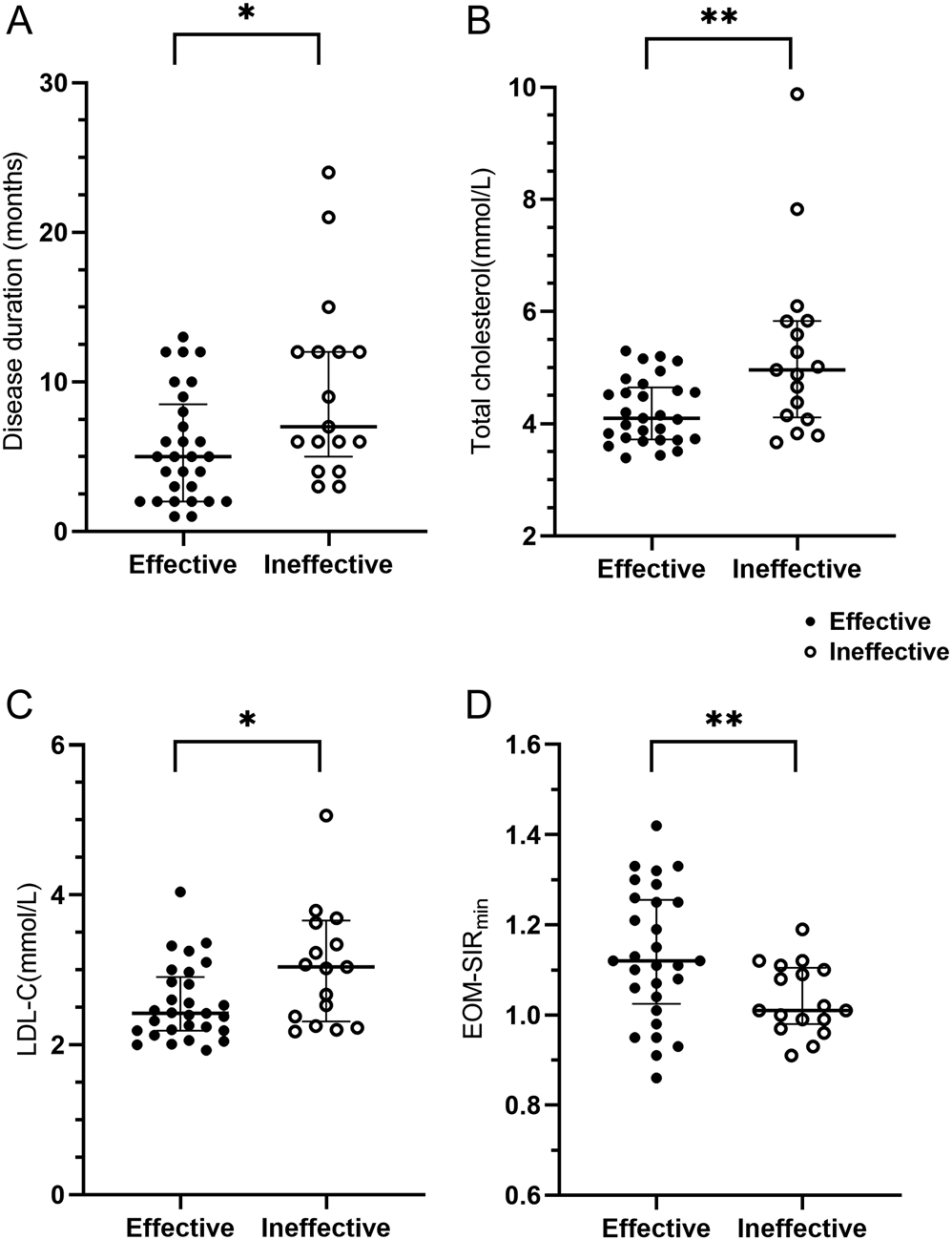
Scatter graphs showing the comparisons of significant clinical and orbital MRI-based parameters between the GC-effective and -ineffective groups. Data are presented using median (center line) and interquartile range (error bars) values. An asterisk represents a signiﬁcant difference (**P < 0.01; *P < 0.05). A. Disease duration in the GC-effective group was significantly shorter than that in the GC-ineffective group (*P* = 0.017). B. Serum TC in the GC-effective group was significantly lower than that in the GC-ineffective group (*P* = 0.006). C. Serum LDL-C in the GC-effective group was significantly lower than that in the GC-ineffective group (*P* = 0.019). D. EOM-SIR_min_ values in the GC-effective group was significantly higher than that in the GC-ineffective group (*P* = 0.005). EOM-SIR_min_, minimal signal intensity ratio of extraocular muscle; LDL-C, low-density lipoprotein cholesterol.

Clinical and demographic details of the study population are presented in Table 1. Patients were previously and currently receiving antithyroid drugs or thyroid hormone replacement therapy based on their thyroid function to maintain euthyroid function (*P* = 0.844). The disease duration of TED patients in the GC-effective group was significantly shorter than that in the GC-ineffective group (5.0 (2.0, 8.5) vs 7.0 (5.0, 12.0) months, *P* = 0.017) (Fig. 3A). As for serological lipid metabolism parameters, GC-effective TED patients showed significantly lower serum TC (4.10 (3.72, 4.65) vs 4.96 (4.12, 5.84) mmol/L,* P* = 0.006) and LDL-C (2.42 (2.19, 2.91) vs 3.04 (2.32, 3.66) mmol/L, *P* = 0.019) levels than GC-ineffective patients, while TG and HDL-C levels did not significantly differ between the two groups (both *P* > 0.05) (Fig. 3B and C).

**Table 1:** Detailed clinical and demographic information of the present study population. The normally distributed numeric data are described as mean ± s.d.,and the nonnormally distributed data are expressed as median (interquartile range).

Variables	Effective	Ineffective	*P*
*n*	29	17	
Age (years)	45.0 ± 12.1	50.6 ± 5.8	0.065
Sex			0.253
Male	12	10	
Female	17	7	
Smoking history			0.181
Yes	8	8	
No	21	9	
History of thyroid medication			0.844
Antithyroid drugs	25	15	
Thyroid hormone replacement	4	2	
Disease duration (months)	5.0 (2.0, 8.5)	7.0 (5.0, 12.0)	0.017^a^
Time since normal range FT_3_ (months)	2.0 (1.0, 4.0)	1.0 (1.0, 3.5)	0.489
BMI (kg/m^2^)	24.2 ± 2.8	24.7 ± 2.6	0.573
Pre-treatment			
FT_3_ (pmol/L)	4.84 (4.44, 5.52)	4.87 (4.35, 5.40)	0.601
FT_4_ (pmol/L)	16.08 ± 3.20	15.66 ± 4.62	0.721
TSH (mIU/L)	0.47 (0.02, 3.70)	0.43 (0.03, 2.88)	0.766
During treatment			
FT_3_ (pmol/L)	4.66 (4.33, 5.36)	5.12 (4.05, 5.38)	0.779
FT_4_ (pmol/L)	15.53 ± 1.85	15.01 ± 3.30	0.553
TSH (pmol/L)	2.12 (1.35, 3.22)	1.59 (0.13, 4.67)	0.600
Post treatment			
FT_3_ (pmol/L)	4.76 (4.41, 5.45)	4.78 (4.20, 5.31)	0.641
FT_4_ (pmol/L)	16.58 ± 2.36	15.48 ± 2.71	0.157
TSH (pmol/L)	1.67 (0.68, 3.29)	1.91 (1.41, 4.20)	0.219
TRAb (IU/L)	3.47 (2.10, 9.06)	3.99 (3.34, 15.89)	0.207
TC (mmol/L)	4.10 (3.72, 4.65)	4.96 (4.12, 5.84)	0.006^a^
LDL-C (mmol/L)	2.42 (2.19, 2.91)	3.04 (2.32, 3.66)	0.019^a^
HDL-C (mmol/L)	1.24 ± 0.30	1.17 ± 0.21	0.418
TG (mmol/L)	1.26 (0.92, 1.60)	1.43 (1.04, 1.77)	0.473

^a^*P* values < 0.05 are considered statistically significant.

BMI, body mass index; CAS, clinical activity score; FT_3_, free triiodothyronine; FT_4_, free thyroxine; HDL-C, high-density lipoprotein cholesterol; LDL-C low-density lipoprotein cholesterol; TSH, thyroid-stimulating hormone; TRAb, thyroid-stimulating hormone receptor antibody; TC, total cholesterol; TG, triglyceride.

Differences in thyroid function before, during, and after treatment between the GC-effective and -ineffective groups were not statistically significant (*P* > 0.05). There was no significant difference in other variables, including smoking history, sex, age, pretreatment BMI, serum TRAb, and the time since normal range FT_3_ between the two groups (all *P* > 0.05) (Table 1).

### Comparison of ophthalmic findings of TED patients between the GC-effective group and -ineffective groups pre- and post-treatment

Table 2 shows that there was no significant difference in ophthalmic performance between patients in the GC-effective and -ineffective groups before treatment. The exophthalmos, eyelid width, and binocular CAS were significantly decreased after treatment in the GC-effective group (all *P* < 0.05), while visual acuity, intraocular pressure, and diplopia were not significantly different between before and after GC therapy. In the GC-ineffective group, the CAS score was also significantly decreased after treatment (*P* < 0.001), but there were no significant differences in other ophthalmic findings between pre-treatment and post treatment (all *P* > 0.05).

**Table 2:** Comparison of ophthalmic findings of TED patients between the GC-effective and -ineffective groups pre-treatment and post treatment.

Ophthalmic findings	Effective (58 eyes)			Ineffective (34 eyes)			*P*^c^
	Pre-treatment	Post treatment	*P*^a^	Pre-treatment	Post treatment	*P*^b^	
Visual acuity	1.0 (1.0,1.0)	1.0 (1.0,1.0)	0.674	1.0 (1.0,1.0)	1.0 (0.8,1.0)	0.834	0.113
Intraocular pressure (mm Hg)	16.6 (15.0, 19.7)	15.7 (14.3, 18.8)	0.167	17.7 (15.2, 22.8)	17.2 (15.5, 22.6)	0.888	0.247
Exophthalmos (mm)	20.0 (18.0, 23.0)	19.0 (17.0, 21.0)	0.040^d^	20.5 (19.0, 24.1)	20.0 (19.0, 24.0)	0.791	0.138
Eyelid width (mm)	10.0 (8.0, 11.0)	9.0 (7.8, 10.0)	0.026^d^	11.0 (8.0, 12.0)	10.5 (8.0, 12.0)	0.669	0.363
Diplopia			0.710			0.618	0.509
Yes	28	26		14	12		
No	30	32		20	22		
CAS	4.0 (3.0,4.0)	1.0 (1.0,2.0)	<0.001^d^	3.0 (3.0,4.3)	3.0 (3.0,3.0)	<0.001^d^	0.591

^a,^^b^Comparisons between pre-treatment and post treatment in the GC-effective and -ineffective groups, respectively; ^c^Pretreatment comparison between the GC-effective and -ineffective groups; ^d^ *P* values < 0.05 are considered statistically significant.

TED, thyroid eye disease.

### Comparison of orbital MRI-based EOM-SIR_min_ values between the GC-effective and -ineffective groups

The quantitative parameter based on orbital MRI findings achieved good to excellent interreader reproducibility (0.718–0.836). Compared to patients in the GC-ineffective group, those in the GC-effective group had significantly higher EOM-SIR_min_ values (1.13 ± 0.15 vs 1.04 ± 0.08, *P* = 0.005) (Fig. 3D).

### Independent predictors of GC effectiveness

Multivariate logistic regression analysis showed that pretreatment serum TC (odds ratio (OR) = 0.253, *P* = 0.015) and EOM-SIR_min_ (OR = 2.036 per 0.1 units, *P* = 0.027) were independent predictors of GC effectiveness in TED patients (Table 3).

**Table 3:** Independent predictors of GC-effective TED

Parameters	*β* coefﬁcient	s.e.	Odds ratio (95% CI)	*P*
TC, mmol/L	−1.375	0.563	0.253 (0.084–0.762)	0.015^a^
EOM-SIR_min (per 0.1 units)_	0.711	0.322	2.036 (1.083–3.826)	0.027^a^

^a^*P* values < 0.05 are considered statistically significant.

EOM-SIRmin, minimal signal intensity ratio of extraocular muscle; GC, glucocorticoid; TED, thyroid eye disease; TC, total cholesterol.

### ROC curve analysis

**Figure 4:**
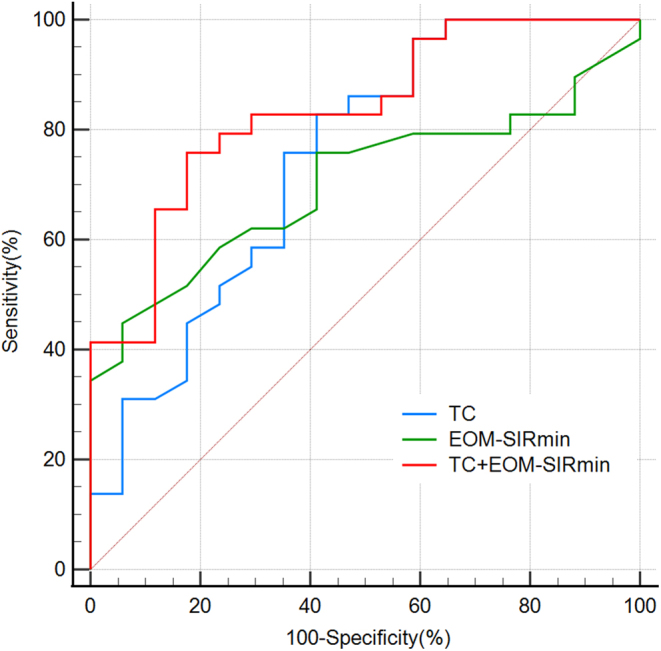
ROC of identiﬁed variables and their integration for predicting GC-effective TED. ROC, receiver operating characteristic; TED, thyroid eye disease; TC, total cholesterol; EOM-SIR_min_, minimal signal intensity ratio of extraocular muscle.

Table 4 displays the detailed ROC curve analysis results. Setting the critical value of TC as 4.8 mmol/L and the critical value of EOM-SIR_min_ as 1.12 resulted in the best prediction efficiency of a single index for GC therapy response (area under the ROC curve: 0.743 and 0.703). In addition, by uniting TC ≤4.8 mmol/L and EOM-SIR_min_ ≥1.12, the predictive power could be further improved (area under the ROC curve: 0.834), with a sensitivity of 75.9% and a specificity of 82.4% (Fig. 4), compared to that attained when using EOM-SIR_min_ alone (*P* = 0.031).

**Table 4:** Predictive performance of identified variables and their integration for GC-effective TED.

Model	AUC	Cutoff	Sensitivity	Specificity	*P*
TC, mmol/L	0.743	≤4.8	82.8%	58.8%	0.151
EOM-SIR_min_	0.703	≥1.12	44.8%	94.1%	0.031^a^
TC+ EOM-SIR_min_	0.834	–	75.9%	82.4%	–

^a^*P* indicated the comparisons between the integrated model and single index models, and values <0.05 are considered statistically significant.

AUC, area under the curve; EOM-SIR_min_, minimal signal intensity ratio of extraocular muscle; TED, thyroid eye disease; TC, total cholesterol.

## Discussion

We identified three main findings in this study, as follows: First, serum TC and LDL-C in the GC-effective group were significantly lower than those in the GC-ineffective group. Secondly, the GC-effective group showed significantly higher EOM-SIR_min_ values than the GC-ineffective group. Thirdly, serum TC and EOM-SIR_min_ were independent predictors of GC effectiveness. Moreover, the integration of TC and EOM-SIR_min_ as two independent predictors could further improve the prediction performance over that of EOM-SIR_min_ alone. Our study demonstrated that the therapeutic effect of GCs in TED patients could be predicted simply and effectively based on readily available serum TC and orbital MRI-based EOM-SIR_min_ values. This observation could help clinicians to individualize the management of TED patients to ensure a better treatment prognosis.

Some previous studies have proven the value of MRI for immunomodulating therapy of TED, including that of some functional MRI techniques (24, 25, 26, 27). Zhai (24) found that T2 mapping could predict the treatment response to intravenous glucocorticoid therapy for TED, while Liu *et al.* (25) found that the pretreatment volumetric T2 relaxation time histogram features of the EOMs could help predict the response to GC therapy in TED patients. However, although functional MRI can provide quantitative metrics, the high variability and low repeatability among different MRI scanners represent marked drawbacks that limit its standardization and wide application. By contrast, the EOM-SIR_min_ used in this study is semiquantitative and easier to apply in clinical practice. The effect of different MRI systems on the metrics would be limited, which is crucial for its wide application in multiple centers. EOM-SIR_min_ has been identified as a potent independent predictor of GC-effective TED (16, 17). However, the minimum SI could be affected by the SI of the fat tissue. If our study could compare T2 mapping with and without fat saturation, it might be better to distinguish the difference in SI and help identify the sources of SI (24). Similar to previous studies, this study found that EOM-SIR_min_ in the GC-effective group was markedly higher than that in the GC-ineffective group. Meanwhile, EOM-SIR_min_ was found to be an effective imaging-independent predictor of GC effectiveness. For the possible mechanism, previous studies believed that the dynamic transition between the active inflammatory phase and the relatively static chronic fibrosis phase of TED may make sense (3). In the acute active phase, with lymphocyte infiltration and secretion of various cytokines, fibroblasts proliferate, orbital tissue exhibits edema, and the volume of EOMs increases (4, 28). Inflammation gradually subsides with the prolongation of the disease course and the evolution of collagen deposition and fat infiltration; then, the ophthalmopathy enters a chronic fibrosis stage (3, 4, 28). EOM-SIR_min_ reflects the most static area inside the muscles whose value was decreased in the GC-ineffective group; this, combined with the accompanied longer disease duration in the GC-ineffective group, may imply an association with the progression of focal fibrosis within the EOMs in this group of patients (16, 17).

Disturbance of lipid metabolism can lead to changes in the body’s homeostasis, which can cause local or systemic chronic inflammation and increase the risk of various diseases like fatty liver, diabetes, and atherosclerosis (10, 29). In recent years, a growing number of studies have noticed the relationship between cholesterol and TED. The 2021 EUGOGO guidelines suggested that high serum cholesterol is an emerging and potential risk factor for TED (6). Previous studies have reported that the presence of TED correlates with hypercholesterolemia in patients with Graves’ disease (12, 13). Researchers have also investigated the link between serum cholesterol and the severity and clinical activity of TED (13). One recent study also found that high baseline serum LDL-C levels may reduce the efficacy of intravenous GC therapy in patients with active and moderate-to-severe TED (14). Similar to previous studies, our study also found that hypercholesterolemia might be associated with the ineffectiveness of GC therapy in TED patients. Hypercholesterolemia was considered to increase the immune process of TED, and accelerate orbital remodeling and fibrosis by inducing a chronic inflammatory state in the body (14). Thus, together with the analysis of EOM-SIR_min_, it is also reasonable that GC-ineffective TED patients had elevated serum TC levels before treatment. These findings are likely to be related to the progression of orbital tissue fibrosis and remodeling, indicating a potential correlation between hypercholesterolemia and GC ineffectiveness in TED. However, serological indicators alone cannot directly reflect the morphological changes in the eye. Therefore, we added objective and individualized MRI parameters which can reflect the structural morphology of the eye, in our study. Moreover, the serological and imaging parameters we selected were relatively simple indicators with strong clinical applicability. In addition, considering that blood lipids are more modifiable than imaging indicators, strengthening blood lipid management before treatment may be helpful in improving GC efficacy. In addition to the above, according to our study results, serum TC >4.8 mmol/L may indicate that GC treatment is ineffective. This suggested that, even within the normal range, relatively high blood lipids may affect the prognosis of TED. Thus, strengthening lipid management may be more helpful to improve the prognosis of the disease. Based on the observation of blood lipids and the efficacy of GC therapy in TED, current studies have suggested that the use of statins may be a factor affecting the efficacy of GC therapy. A randomized controlled trial previously demonstrated that the addition of statins to GC treatment could improve disease outcomes of TED patients with hypercholesterolemia (30). In the pathogenesis of TED, orbital fibroblasts are the targets of the autoimmune attack (31). Statins could inhibit orbital tissue fibrosis by inhibiting the differentiation of orbital fibroblasts into myofibroblasts induced by transforming growth factor-β (TGF-β) (32). Additionally, statins could also affect orbital tissue remodeling by downregulating the expression of adipogenesis genes and inhibiting adipogenesis in preadipocytes and fibroblasts (33, 34, 35). In this paper, we found that high serum cholesterol levels might predict the ineffectiveness of GC therapy in TED patients. We believe that both these studies indicate that lipid profile might be an important mechanism and also a therapeutic target in patients with TED. The above discussion also clarified the relationship between serum cholesterol and GC treatment effectiveness in TED patients from another perspective.

Clinically, both sensitivity and specificity were indispensable for predicting GC therapy response in TED patients. High sensitivity could improve the diagnostic ability of GC-effective TED, while high specificity could increase the diagnostic confidence of patients who are unresponsive to GCs. GC-effective patients could be treated with intravenous glucocorticoids, and other immunosuppressive therapies or surgery could be selected for GC-ineffective patients. In our study, serum TC and EOM-SIR_min_ were identified as independent predictors of GC-effective TED. We found that the integrated prediction model of serological and imaging parameters had better prediction performance than the single imaging prediction model. By specifically uniting TC ≤ 4.8 mmol/L and EOM-SIR_min_ ≥ 1.12, GC-effective and -ineffective TED could be distinguished from each other effectively, with a sensitivity of 75.9% and a specificity of 82.4%. The finally constructed model provided a novel, simple, and effective approach for early prediction of GC therapy response in patients with active and moderate-to-severe TED. Our results should contribute to individualized treatment of TED and improve patients’ quality of life and disease prognosis.

Some limitations were present in this study. First, this was a retrospective study with a limited sample size conducted at a single center, which could lead to potential selection bias. Further multicenter prospective studies with larger sample sizes are needed to confirm our study results and clarify the optimal threshold value. Secondly, pathological evaluation of orbital tissues could not be achieved given the retrospective nature of our study. Thus, further research matching the pathological and imaging changes in TED patients would be valuable. Thirdly, SIR_min_ in our study was measured based on one kind of 3.0-Tesla MRI system (Magnetom Skyra; Siemens Healthcare). The SIR may be influenced by scanners or imaging parameters (e.g., field strength and echo time). Therefore, the provided cutoff values obtained at other centers and scanners may be different. Moreover, our MRI data were obtained based on a single center’s equipment, which may have the problem of popularization when applied to other manufacturers and machines. The real value in quantitating the changes of parameters, the cost and availability remained controversial. In the future, multicenter data should be collected and individualized threshold values should be established based on multiple types of MRI equipment. Additionally, future studies incorporating T2 mapping-derived quantitative parameters may correlate better with the pathology and further help to improve the predictive efficacy to GC.

Our study indicated that serum TC and orbital MRI-based EOM-SIR_min_ could be conducive to predicting the effectiveness of GC. Integrating serological lipid metabolism and orbital MRI might be a promising approach for improving the predictability of treatment response, thereby subsequently assisting in making individualized treatment plans for patients with active and moderate-to-severe TED.

## Declaration of interest

The authors declare that there is no conflict of interest that could be perceived as prejudicing the impartiality of the study reported.

## Funding

This work was supported by the National Natural Science Foundation of Chinahttp://dx.doi.org/10.13039/501100001809 (NSFC) (No. 81801659) to HH, “Thyroid Research Program of Young and Middle-aged Physicians” from China Health Promotion Foundationhttp://dx.doi.org/10.13039/501100017661 (2019 (PB19)) to HC, Research Foundation of Yili Institute of Clinical Medicine (yl2021ms03) to HC, Jiangsu Provincial Medical Key Discipline (Laboratory) ZDXK2http://dx.doi.org/10.13039/50110001023802202 and Jiangsu Province Hospital (the First Affiliated Hospital with Nanjing Medical Universityhttp://dx.doi.org/10.13039/501100007289 Clinical Capacity Enhancement Project (JSPH-MC-2021-8)).

## Statement of ethics

This study was conducted in accordance with the Declaration of Helsinki and approved by the Ethics Committee of the First Affiliated Hospital of Nanjing Medical University (No. 2019-SR-416). The informed consent requirement was waived by the Ethics Committee of the First Affiliated Hospital of Nanjing Medical University due to the retrospective nature of the study.

## Author contribution statement

HZ, HH, XX, and HC designed the study, supervised the data collection process, checked the final analysis results, and revised the manuscript. HZ, YW, and XD collected the data, carried out the initial analysis, and drafted the initial manuscript. HZ, LC, JZ, and WC collected the data, completed the analysis and interpretation of the data, and participated in the drafting of the initial version of the manuscript. HZ, HH, XX, and HC checked the analysis results and reviewed the manuscript.
